# Ethical Resource Allocation in Policing: Why Policing Requires a Different Approach from Healthcare

**DOI:** 10.1080/0731129X.2024.2327819

**Published:** 2024-04-04

**Authors:** Hannah Maslen, Colin Paine

**Affiliations:** Oxford Uehiro Centre for Practical Ethics, University of Oxford; Thames Valley Police, UK

**Keywords:** Policing, ethics, resource allocation, distributive justice, healthcare

## Abstract

This article examines the inherently ethical nature of resource allocation in policing. Decision-makers must make trade-offs between values such as efficiency vs. equity, individual vs. collective benefit, and adopt principles of distribution which allocate limited resources fairly. While resource allocation in healthcare has been the subject of extensive discussion in both practitioner and academic literature, ethical resource allocation in policing has received almost no attention. We first consider whether approaches used in healthcare settings would be suitable for policing. Whilst there are some high-level similarities in the relevant ethical considerations and processes—such as aiming for efficient and equitable resource deployment—there are also fundamental differences between the two settings, which we argue necessitate a different approach. These differences include: (1) greater diversity and incommensurability of the benefits of policing, (2) more frequent non-linearity in returns on policing investment, (3) greater “ethical divisibility” (permissible scaling down) of programs in policing, (4) a requirement to assess “need” for policing resources at the collective (rather than individual) level, and (5) clearer primacy of equity considerations in policing. Having drawn out the implications of these differences, we sketch some tentative proposals for ethical resource allocation in policing.

## Introduction

Imagine having to make the following decision:

You are the Chief Constable of a police force under significant financial pressure. Your budget requires further cuts and you must decide from which areas to make significant savings. You consider the following aspects of current performance and demands:

- Your community complains that they do not see enough officers on patrol and the local media is running a campaign called “save our PCSOs (Police Community Support Officers).”
- Your force’s emergency response times are deteriorating due to prior cuts to the number of officers.
- Increases in sexual offences means that the investigations by CID are taking longer, resulting in criticism from vocal local domestic abuse advocates.
- A recent incident involving a high-risk missing child high-lighted the fact that the force has too few school officers, and the leader of the council has asked to meet you to discuss what you propose to do.
- Child abuse investigation is under resourcing pressure and many officers are taking sick leave owing to stress and burnout.
- A petition has been handed in indicating that a local community is deeply unhappy with the recent closure of their police station front counter to make savings.
- The force is doing well with its investigation of serious organized crime with a series of recent successes in recovering guns, but any reduction in this team could result in increased murders by criminal gangs.

You must make additional, ethical cuts somewhere, knowing it will compromise some service area. Where will you reduce your force?

This example might seem exaggerated, but making these decisions is normal business for Chief Constables.^1^ Yet, there is no systematic framework to assist with enumerating and weighing up the ethical considerations. Should you act primarily on the loudest pleas from your local communities, especially if this has implications for public trust? Should you focus more on high-harm or high-volume issues? How much should officer wellbeing matter?

Decision-making about resource allocation in both policing and healthcare is inherently ethical in nature. The decisions made result in significant benefits and harms (or failures to avoid harms) for those affected; some harms can be catastrophic, yet, with limited resources, not all benefits can be achieved, nor all harms prevented. Deciding which interventions should receive funding and which should be lower priority requires making trade-offs between values such as efficiency and need, between individual and collective benefit, and determining general principles of distribution that achieve the fairest allocation of limited resources.

Resource allocation in healthcare has been the subject of much discussion, and numerous proposals have been put forward for the most ethical decision-making strategies. In practitioner settings, Programme Budgeting Marginal Analysis has been explored,^2^ which incorporates and draws on similar economic principles to those governing the narrower, Quality-Adjusted Life Year (QALY) approach to allocating resources. In academic discussion, the limitations of QALY-based approaches have been well rehearsed, and writers have discussed various ways in which allocation should be more sensitive to equity considerations.^3^ To-date there has been far less systematic discussion of *ethical* resource allocation in policing, both in practitioner and academic forums.

In academic policing ethics, discussions often revolve around issues of misconduct, corrupt culture, and police use of force.^4^ However, it is crucial to recognize that resource allocation within policing is an equally significant ethical issue. While misconduct, culture, and use of force directly impact police legitimacy and effectiveness,^5^ the allocation of resources holds comparable importance as it affects the entire spectrum of policing activities. Yet, in practice, it has been reported that “Forces have not fully utilized looking at other sectors for inspiration and guidance for allocating resources. More detailed investigation of the Cost-Effectiveness Analysis (CEA) model utilized in the health sector may provide some useful insights.”^6^

In this paper, given the paucity of literature on the ethics of resource deployment in policing, we examine whether approaches used in healthcare settings would be fit for purpose for policing. We argue that, although some very general principles for ethical resource allocation transfer across to policing—including with respect to processes—there are some fundamental differences between the two settings that require police decision-makers to pursue a different approach. Key differences include: (1) greater diversity and incommensurability of benefits in policing; (2) greater likelihood of non-constant returns to scale (non-linearity of marginal benefits) in policing; (3) greater “ethical divisibility” (permissible scaling down) of programs in policing; (4) assessment of “need” for policing resources at the collective rather than individual level; and (5) clearer primacy of fairness considerations in policing. Having explicated these differences and their significance for resource allocation, we finish by sketching the rough shape of a decision-making framework for policing. We intend this sketch to be a starting point for further development.

## Policing is in Urgent Need of Principles to Guide Ethical Resource Allocation

Over the last decade senior UK police chiefs have faced difficult decisions on the deployment of officers driven by the loss of 20,600 officers during the period of austerity.^7^ At the same time demand on the service has increased in volume and complexity.^8^ Consequently, policing is failing to meet demand effectively.^9^ Unpalatable decisions have to be made as to what functions should be prioritized, and as a necessary consequence, which other functions should be deprioritized in the face of increasing demand.^10,11^ There is no single response from forces to this challenge; some have chosen to reduce PCSOs (the visible face of policing),^12^ some have reduced the provision of police station front counters,^13^ some have reduced the numbers of officers in neighborhood policing,^14^ and others have reduced their investigative capacity.^15^

These resource decisions involve trade-offs. For example, maintaining investment in counter terrorism policing has meant reductions in trustbuilding neighborhood policing in some forces. Chief officers stated that they must place “urgent threats” above “nice to do” activities such as visible patrolling of the streets or visiting all victims of crime.^16^ As the chair of the police Federation said, “We can’t do everything—there are going to be situations where we simply can’t deliver the policing we want to deliver.”^17^ It is not possible to meet all public need for policing and therefore decisions must be made which impact both individuals and communities.

Resourcing challenges also arise during periods of growth. The government’s pledge to increase the number of police officers by 20,000 in 2019 (largely reversing the previous decade of cuts to numbers) resulted in new challenges affecting decisions about which policing functions should benefit.^18^ There is a need for more visible community policing, the ability to investigate serious crime by CID, the need to tackle online pedophile activity, effective enforcement of traffic legislation to reduce road deaths, better safeguarding of individuals at risk of domestic abuse, investment in officers to manage high threat sexual offenders, police efforts to tackle corruption and better investigation of child sexual exploitation. In light of these issues, how should chiefs make resourcing decisions? These decisions in periods of growth carry the same ethical complexity as those in times of austerity.

Even in the absence of changes to the number of officers, there remain ethical challenges as to how to deploy a finite number of officers to an almost inexhaustible demand for policing.^19^ Chiefs must consider how much resource to deploy to crime prevention, such as neighborhood policing, versus the investigation of crimes after the event, such as CID.

Equally they must decide how much resource to put into tackling high-volume low-impact crimes such as vehicle crime versus low volume but high impact crimes such as murder. As the crime and policing landscape changes, so chief officers must continually review and reconsider their resource deployments.

It is important that we make the decision-making context clear at the outset: our focus in this paper is on how individual police forces do and should allocate the limited resources allotted to them from the public purse. The granularity of the decision-making, then—as a function of the structure of police forces in the UK (with similar parallels to jurisdictions in the U.S. and Australia)—is at a *regional*, rather than national level. This is in some contrast to the granularity of the allocation of healthcare resource decision-making, which often occurs at the national level, at least in the UK. Further, we are examining how police forces should allocate the limited resources that they are given, rather than asking how much or how little should be spent by the government on the police service. Our paper therefore does not seek to engage with the question of whether the police service should receive more or less public money.

It is also important to acknowledge that in both healthcare and policing, there will be some nationally formulated priorities, which put some constraints on the discretion that decision-makers have when allocating their budgets. In policing, these come in the form of statutory obligations, government targets, and directives from the National Police Chief’s Council. However, the Chiefs of the various police forces will have some discretion regarding how to comply with these national priorities.

In addition to potential differences in how Chief Constables comply with national directives, there is evidenced inconsistency in resource deployment between the several UK forces for the portions of the budgets over which they have full discretion. HMICFRS’s “Value for Money Profiles” allow comparison on resource allocation per capita, revealing significant differences in spend for the same functions, like neighborhood policing or investigations.^20^ Inconsistency in decision-making could indicate that some deployments are more ethically justified than others, however, there may be justified inconsistency given that different forces will have different service demands and be facing different challenges. In the next section, we explore current decision-making processes, noting the absence of a systematic framework.

## Current Allocative Decision-Making in Policing Does Not Engage Sufficiently with the Ethical Considerations

The absence of clear ethical resource allocation decision-making by chief officers results in more informal “rationing” decisions being made by officers “on the ground” through informal heuristics, such as those we outline below.^21^

### “It’s all economics—just do what’s most efficient”

It is often suggested that police resourcing is a matter of simply maximizing the efficiency of deployment or adopting effective demand reduction strategies.^22^ However, whilst reducing preventable demand supports benefit maximization by freeing up resources to be utilized elsewhere, it cannot resolve difficult choices about how to re-allocate this resource.

### “We police by consent, so we should just ask our communities what they want”

Others suggest that ethical resource allocation should represent the needs of the communities served to build public trust. However, simply engaging with communities is not an automatic shortcut to ethical resource allocation. Communities can overlook unseen policing demands like child abuse, domestic abuse, organized crime, and threats like terrorism. They may ask the police to focus on more visible nuisance behaviors such as drunkenness or anti-social behavior. This risks meeting the needs of a vocal minority but overlooking the vulnerable without a public voice.^23^ Responsiveness to communities is critical to police legitimacy but does not ensure ethical resource use.

### “Officers over admin—send resources to the frontline actually tackling crime, rather than to desks”

The notion of preserving the policing “front line” by cutting “back office” expenses oversimplifies resource allocation. Definitions of these terms are unclear;^24^ and modern police forces are intricate systems with interrelated roles. Reductions in areas like intelligence, analysis or supervision can result in inefficient or counterproductive frontline deployment. The back office often enhances frontline efficiency; and some forces have begun putting officers back into the “back office” for this reason.^25^

### “Cut everything equally, because that’s the fairest”

The “salami slicing” approach, making equal cuts or growth across all functions, is simple and attractive but likely to be ethically sub-optimal. It disregards the efficiency and equity of each function, solidifies existing resource issues by “baking in existing resource allocation issues,” and neglects that some functions handle cuts better than others.

### “One thing at a time—let’s not overcomplicate”

The usual police resource allocation approach considers one issue at a time, often due to criticism or incidents. While addressing specific shortfalls, this isolated approach can cause siloed thinking. Without comparing resource needs across departments, inappropriate investment may occur. Evaluating each decision separately can miss the inevitable trade-offs between resource investments in one area and cuts in another, leading to suboptimal resource deployment.

### “We know what’s ethical, just follow the Code of Ethics”

The College of Policing’s Code of Ethics (2024) is designed to put ethics at the core of police decisionmaking. It outlines three overarching values—”Courage,” “Respect and empathy,” and “Public service”— and 15 sub-principles like “being fair and impartial,” and “responding to individual needs.” While these principles can broadly support resource allocation decisions, principles such as “being fair and impartial” do not provide sufficient guidance for a senior officer deciding, for example, whether to prioritize resources for child abuse investigation or neighborhood policing. In short, the Code is not designed to tackle the complexity of resource allocation.

Given the lack of systematic principles employed in practice, we can ask whether there are any existing academic proposals for a resource allocation framework. In the next section we survey the sparse literature to demonstrate that this question has barely been raised, and that no comprehensive proposals currently exist.

## What Does Existing Literature Say About Resource Allocation Decision-making in Policing?

How resources *are* allocated in policing has been explored in the literature, although little attention has been given to discussing how resources *should* be allocated. Ludwig et al. conducted interviews with PCCs and Chief Finance Officers in police forces in England and Wales, finding that resource allocation is primarily driven by local demands and priorities, with limited long-term planning.^26^ Ludwig et al. note that little cost-effectiveness or cost−benefit analysis is conducted within forces, but, although they recognize the potential value of such analysis, they do not propose specific methods for implementing it.

Zhuang and Collier investigated resource allocation methods employed by a police department to combat serious and organized crime, concluding that decision-makers employ a combination of “rational” and “non-rational” approaches.^27^

Several papers have proposed quantitative approaches to resource allocation in policing. However, these approaches often focus on specific areas of decision-making and do not consider the ethical dimensions of resource allocation. Shumate and Crowther propose how queuing theory can determine the most efficient allocation of officer hours for responding to events.^28^ Den Heyer examines economic modelling of strategic police allocation during times of austerity, highlighting the need for a process based on social, demographic, and strategic information.^29^ Although these approaches address efficiency in officer allocation, they do not account for the complex trade-offs involved in allocating resources across different areas of policing.

Farmer develops an economic theory of police resource allocation, distinguishing between “altruistic objectives” focused on maximizing social welfare and “bureaucratic objectives” driven by purposes other than the public interest.^30^ Farmer emphasizes the multi-layered nature of decision-making in policing and the influence of political factors but does not propose specific allocation methods.

There are only a few papers explicitly addressing the ethical dimensions of resource allocation in policing. Charman and Williams analyze qualitative data on the barriers faced by rape complainants in accessing police resources, highlighting unfair distribution driven by officers’ (mis)-perceptions of “deservedness.”^31^ However, their focus is on inequitable access to processes (and unethical dimensions of these processes) rather than on force-wide funding allocation. Wheeler examines ethical trade-offs involved in allocating officers to crime hot spots, considering the balance between crime reduction and reducing racial disparities in stops and arrests.^32^ While this paper provides insights into ethical trade-offs, it is limited to a specific resourcing activity and focuses on distribution of the *burdens* of police activity.

Carr-Hill provides perhaps the most detailed and relevant proposal to-date.^33^ He highlights that “the fundamental problem for developing a robust resource allocation formula in any sector is to develop a model which links “need” for resource to the appropriate allocations.”^34^ Carr-Hill proposes a formula for resource allocation, suggesting that the variation in “concern about crime” across geographic areas could serve as a proxy for estimating the level of need for policing resources. Although this approach considers ethical considerations and fairness in allocation, it does not provide a comprehensive framework for ethical resource allocation which also factors in efficiency and the relative public value of the activities funded. Additionally, the subjective nature of the measure of “concern about crime” raises concerns about potentially shifting resources to those who voice complaints rather than those truly in need. We do, however, agree both that a measure of need is central to a policing resource allocation framework, and that the appropriate unit of analysis for assessing need for policing resources is at a collective, rather than individual level.

Given the scarcity of existing proposals, we now turn to consider what might be learnt from approaches employed in healthcare. We start by highlighting the way in which similar trade-offs arise in both settings, and outlining the approaches used in healthcare.

## What Approaches are Used in Healthcare?

To address the need for allocation principles, policing could draw insights from discussions and practices in healthcare. Both sectors face the challenge of balancing the goal of maximizing total expected benefits (efficiency) with allocating resources based on deservingness or strength of claim (equity). In healthcare, prioritizing resources based on severity of need (e.g. sickness) may result in allocating substantial resources for small benefits, while allocating the same resources to less severe cases could generate greater overall benefit.

In the context of policing, there are also situations where efficiency and equity conflict. While addressing need often aligns with generating the most value for money, there are cases where addressing the most severe needs may be more costly or less beneficial from a purely cost-effectiveness perspective. For example, chief officers may need to decide whether to invest more resources in preventive measures for low level sexual offenses, which offer a higher return on investment, or allocate resources to the investigation of individual rape cases with vulnerable victims, even if charges are unlikely. Similarly, they may face the choice of investigating easier-to-solve acquisitive crimes over difficult cold case murders. These examples highlight the challenges faced by chief officers in balancing the response to serious vs. voluminous crimes and considering the trade-off between need and efficiency.

In healthcare, various proposals have been suggested to guide decision-making in navigating these allocative dilemmas. One such approach is Programme Budgeting and Marginal Analysis (PBMA), derived from economic principles.^35^ PBMA emphasizes the importance of opportunity costs and marginal gains/losses, taking into account multiple criteria such as need, efficiency, equity, and core purposes. By incorporating techniques like multi-criteria decision analysis (MCDA), PBMA allows for the identification, weighing, and trading-off of relevant criteria, resulting in cost–benefit ratios for different areas of spending that can inform resource allocation decisions.

### The key stages of PBMA in healthcare:^36^

- Determine the aim and scope of the exercise
- Compile a program budget (map of current activity and expenditure)
- Form marginal analysis advisory panel and stakeholder advisory groups
- Determine locally relevant decision-making criteria with input from decision-makers and stakeholders (e.g. service providers, patients, public)
- Advisory panel identifies options in terms of (1) Areas for service growth (“wish list”); (2) Areas for resource release through producing the same level of output (or outcomes) but with fewer resources; (3) Areas for resource release through scaling back or stopping some services (“shift list”)
- Advisory panel makes recommendations in terms of (1) Funding growth areas with new resources; (2) Decisions to move resources released through increased productivity to areas of growth; (3) Trade-off decisions to move resources from one service to another if relative value is deemed greater
- Validity checks with additional stakeholders and final decisions to inform budget planning process

PBMA is most often used to make decisions about whether or not to fund *new* programs (albeit against the backdrop of the potential to shift resources from existing programs). However, it has been proposed that PBMA can also be used as a tool to assist with disinvestment decisions,^37^ and the MCDA component is particularly suited to formalizing how service areas compare in the extent to which they deliver benefits for units of cost— essential information for disinvestment decision-making. Proponents claim that MCDA can assist with “identifying the ‘least worst’ options from which to disinvest.”^38^

Although the general approach of PBMA could be applied to policing, we will argue that key features will not be directly transferable to policing. The use of QALYs to estimate cost-effectiveness ratios is one of these features. We provide an overview of QALYs, before moving on in the following sections to examine what can and what cannot be transferred to policing from resource allocation approaches in healthcare.

### Overview of QALYS

QALYs are a widely used measure in health economics to assess the effectiveness and value of healthcare interventions. A QALY is a unit of measurement that combines both the quantity and quality of life lived. It is calculated by multiplying the time spent in a particular health state by the quality of life experienced during that time. The quality of life is assessed using a preference-based measure, such as the EuroQol 5-Dimension (EQ-5D) questionnaire, which asks individuals to rate their health state on several dimensions, including mobility, selfcare, and pain/discomfort.

When decisions are being made about funding different healthcare programs, QALY league tables rank different procedures in terms of their marginal costs per QALY gained. Interventions with low cost per QALY gained ratios are placed, in order, at the top of the league table and those with higher ratios at the bottom. Such a listing implies that a higher priority should be assigned to those procedures closer to the top of the list. Procedures with a lower cost per QALY ratio should, in theory, receive resources at the expense of those with higher ratios, in order that more QALYs overall will be produced for the available resources.

There are numerous technical and ethical problems with the QALY approach, which we will not rehearse here.^39^ For the purpose of this paper, we do not need to provide an argument in support or in opposition to the use of QALYs in healthcare, as we will argue below that, even if they were suitable as an approach in healthcare, they would not be suitable for use in policing.

## What is Transferable to Policing?

In this section, we identify what can and should be transferred from approaches in healthcare. There are some general principles which guide resource allocation in healthcare that will be applicable to policing at the macro level. As noted above, in broad strokes, considerations of *efficiency* and *equity* (or fairness) will be as relevant to resource allocation in policing as they are to healthcare. Efficiency is relevant because it affects the overall amount of benefit that can be generated; equity is relevant because it concerns the distribution of benefits among individuals. However, as we will argue, *how* these principles are operationalized will not be transferable, due to differences between the goals and decision-making contexts of the respective sectors. We also note transferable process-related principles relating to the *transparency, accountability* and *monitoring* of the decisionmaking process.

### Principle 1: Efficiency (or, “Cost-effectiveness”) of Resourcing is a Relevant Ethical Consideration

Although we have argued that resource allocation is ethically complex in part because it requires considering factors *in addition to* efficiency, it should be obvious that we are not claiming that efficiency is irrelevant, nor that efficiency is not itself an ethical consideration.^40^ Clearly, there is an ethical imperative not to “waste” limited public money, and foreseeable failures to generate benefit when these resources could be used to generate benefit would be unethical. For example, having unnecessarily complex internal processes necessitating duplication which require a resource intensive level of administrative support would be wasteful. In healthcare, allocating resources efficiently can help ensure that patients receive the best possible care with the available resources. This could, for example, involve prioritizing funding of treatments that are most cost-effective for a particular condition. Avoiding straightforward inefficiencies *where this is not in defensible service of promoting some other value* will similarly apply as an ethical principle of relevance to resource allocation in policing.

Cost-effectiveness is relevant to fair resource allocation but does not exhaust the relevant ethical considerations: i.e. allocation that is *only* sensitive to cost-effectiveness will not necessarily (and is very unlikely to) produce the fairest distribution. Vice versa, allocation that is *not* sensitive to cost-effectiveness will also fail to achieve the most ethical use of resources. Cost effectiveness is relevant to, but not determinative of, ethical resource allocation.

### Principle 2: Equity, or Responding to “Need,” Will Also be Relevant

The ethical consideration of “need” is incorporated into some approaches to resource allocation in healthcare. Programme Budgeting Marginal Analysis, for example, includes “equity” as a consideration that should feature in the multi-criteria decision analysis. Pure benefit maximization can have the consequence that resources are directed to those who are already relatively well off, in this case in terms of health, as the severity of the illness is not taken into account when assessing the magnitude of improvement.

Equity in healthcare resource allocation refers to the concept of fairness in the distribution of healthcare resources.^41^ It is synonymous with the notion of “fair shares” in the allocation of, and “fair opportunities” in access to, healthcare resources and services. Crucially, equity does not necessarily require *equal* shares or *equal* opportunities, as this is not always fairest.

There are two dimensions of equity: horizontal equity and vertical equity. When horizontal equity is achieved, people in the same circumstances or with the same health needs are treated equally, with the same access to the same services. This serves to avoid discrimination and preferential treatment. Vertical equity, on the other hand, requires that people in relevantly different circumstances or with different health needs are treated differently, usually with those in greater need receiving more resources. This aims to redistribute resources and achieve fairness, with the core idea being that some individuals—for example those in greater need—have a greater claim on resources.

Resource allocation in policing should also be subject to guiding considerations relating to “need” or relative claims on resources. If need were not taken into account, then allocation purely based on benefit maximization could have the consequence that policing would favor low-seriousness crime prevention activity which could benefit thousands, over the investigation of a very difficult-to-solve murder. But this would leave indefensible gaps in the service to the most vulnerable populations. As in healthcare, fairness matters alongside cost-effectiveness. Indeed, the World Health Organisation (WHO), whilst recognizing that cost-effectiveness will also govern resource allocation, sets out the guiding consideration of “Fair distribution: Coverage and use of services should be based on need, and priority should be given to the policies benefiting the worse off groups.”^42^

In addition to these two transferable substantive ethical considerations, there are also principles relating to processes, which have an “ethical” dimension in so far as they ensure good practices, and so support pursuit of principles one and two. We briefly note three process-related principles which we assume to be uncontroversial, so although worth noting, we do not argue for them at length.

### Principle 3: Decision-making Should be Transparent—Communicating What and How Decisions are Made

Transparency in PBMA involves openly disclosing information on the decision-making process, criteria, and rationale for resource allocation. This transparency helps stakeholders and the public understand why certain programs receive more funding. Resource allocation in policing should be similarly transparent and is crucial for maintaining legitimacy. However, due to security concerns, some information may need to remain confidential. For example, forces may not wish to reveal how many covert surveillance assets they have as this information could be used by criminals. Nevertheless, redactions should be justified internally.

### Principle 4: Decision-Makers Should be Accountable for Resource Allocation Decisions Made

PBMA incorporates accountability measures like audit trails and validity checks, ensuring decision-makers are held responsible for resource allocation choices. In policing, accountability mechanisms should be integrated into resource allocation processes. Holding decision-makers accountable enhances legitimacy and fosters a culture of learning and innovation. While policing has existing accountability structures, making resource allocation a regular agenda item should be normalized, ensuring ongoing scrutiny and transparency.

### Principle 5: Outcomes Should be Monitored and Decisions Reviewed

In addition to accountability mechanisms encouraging a culture of learning, there should also be active steps taken to formally evaluate the decisions made. In healthcare, PBMA encourages the ongoing monitoring and evaluation of program performance against the allocated resources. If a program fails to achieve the desired outcomes, decision-makers may need to reassess their choices in subsequent budget cycles. This duty to selfreview is intended to ensure that resources are not wasted. Similar evaluative practices should be adopted for resource allocation decisions in policing.

## What Differences Preclude the Adoption of Healthcare Approaches?

We now turn to examining differences between healthcare and policing, which necessitate a different approach to resource allocation.

### Difference One: The diversity and incommensurability of policing benefits makes their measurement far more complex than measuring benefits in healthcare

As explained above, although benefit maximization does not exhaust the principles guiding ethical resource allocation, it is nonetheless a central, and ethically relevant consideration. If all else were equal, public money should be spent in a way that produces the most benefit to the public as possible. Although this is equally true of policing resource allocation as it is of healthcare, the way that benefits are measured will differ. We will argue that it is not only the nature of the benefits that differs between healthcare and policing, but also that, whilst healthcare benefits can arguably be (and in practice are) compared on one metric, this is not possible for policing.

As described above, the benefits of healthcare programs and interventions are usually conceived as some composite of improvements relating to length and/or quality of life of individuals. Regardless of the preferred specification of these aspects of healthcare benefit, most practical applications proceed on the basis that programs can be compared using the same metric. For example, let’s consider the following two, very different, healthcare programs and how they can be compared in terms of QALYs gained (or prevented from being lost):

(1) Program One: A smoking cessation program that targets heavy smokers who have been smoking for many years. This program involves personalized counselling and the use of nicotine replacement therapy. The goal of the program is to help participants quit smoking and reduce their risk of developing smoking-related illnesses.
(2) Program Two: A selective serotonin reuptake inhibitor (SSRI) for individuals with depression. This medication alleviates the symptoms of depression. The goal of the medication is to improve the mental health and well-being of patients and prevent the negative outcomes associated with depression, such as suicide and decreased quality of life.

These interventions have different purposes but can still be compared in terms of their cost-effectiveness using QALYs. For example, we can estimate the number of QALYs gained from the smoking cessation program by estimating the reduction in the risk of smoking-related illnesses and the impact on longevity, as well as any improvement in quality of life. Similarly, we can estimate the number of QALYs gained from the SSRI medication by estimating the reduction in depressive symptoms and the resulting improvement in quality of life, as well as impacts on longevity as a consequence of reducing the incidence of suicide. By comparing the cost of each intervention to the estimated number of QALYs gained, we can determine which intervention is more cost-effective.

In emphasizing the relative complexity of measuring the benefits of policing, we acknowledge that the QALY approach to measuring the benefits of healthcare is also likely to be an oversimplification. That is, it may be the case that policing and healthcare benefits are equally complex. Indeed, improved health can have a range of benefits not accounted for by a measure that captures only length and quality of life. Better health is associated with better educational and economic outcomes, for example. However, such indirect benefits and positive externalities are achieved, *via health*, for which length and quality of life is a plausible, if simple, proxy. So, even though QALYs do not capture all the wider benefits that accrue from healthcare interventions, they do offer an operationalization of the *primary goal*.

It should further be noted that not all benefits associated with either policing or healthcare will necessarily be relevant to an estimation of the benefits that they produce for resource allocation purposes. Only those benefits with a link to their remit and goals will be relevant. If this were not the case, then all public services would have the task of simply producing the most benefit, as pure utility maximizers, which may involve spending money on activities completely unrelated to their purposes and remits. While it might be true that the healthcare service indirectly benefits the economy, for example, economic prosperity is not and should not be the primary goal of the healthcare service. If such benefits are achieved via good health, this is a happy by-product, but it is still good health that is the primary goal. That a benefit is produced, does not necessarily mean it is to be factored in to how resources should be spent. This is determined by remit and primary goals, otherwise public services have to consider all possible benefits and become pure utility maximizers.

In contrast to healthcare, the primary benefits of policing activities (and even less so their indirect benefits and positive externalities) are not so easily reducible to one concept. Being so, comparing the benefits of policing programs on a single metric analogous to QALYs is much less conceptually plausible. For example, the benefits of preventing a spate of burglaries (rights protections, wellbeing protection) are difficult to compare with the benefits of an investigation and conviction (criminal justice value), which are different in turn from the benefits of promoting public trust and confidence in the police service or reducing the fear of crime. But all of these are *primary goals* of policing activities. Policing has more numerous and more diverse primary goals than does healthcare. It could be argued, as it might by a utilitarian, that at the most fundamental level, these benefits are all a matter of increased (or protected) wellbeing, and so programs could, at least theoretically, be directly compared on this metric. We see theoretical and practical difficulties with this view.

From a theoretical point of view, the goals of policing are foremost (although not exclusively) concerned with preventing rights infringements and supporting justice, rather than maximizing wellbeing, even though preventing rights infringements will often have consequences for wellbeing. The College of Policing’s *Policing Vision (2025)* sets out that “The mission of the police is to make communities safer by upholding the law fairly and firmly; preventing crime and antisocial behavior; keeping the peace; protecting and reassuring communities; investigating crime and bringing offenders to justice.”^43^

Indeed, a rights-based account of the justification of policing, such as that developed by Seumas Miller and colleagues,^44^ could emphasize that certain benefits of policing programs and interventions cannot be reduced to improvements in wellbeing, but rather are grounded in the value of protecting individual rights and freedoms. For example, the deterrence of crime through visible policing may prevent violations of rights such as property rights or the right to personal safety. Criminal investigation is necessary to promote the institutional rights of victims to due process and justice and will often also have further rights-protecting effects, via general deterrence.

This said, not all benefits of policing interventions and services are directly connected to protecting human and institutional rights. Initiatives to reduce fear of crime, for example, may more straightfor-wardly be seen as aiming at improving quality of life (wellbeing) by reducing aversive psychological states. Nevertheless, the diversity of benefits of policing, some of which are more plausibly understood in terms of the protection of moral rights, and others more directly connected to wellbeing, make a single metric of benefit conceptually incoherent.

Further, even if an attempt were made to create a composite measure of benefit that took into account relative importance of rights protections *and* effects on quality and length of life (i.e. wellbeing), there are significant practical difficulties in determining the individuals to whom the benefits of policing accrue, and in what time frames. In contrast to healthcare, determining who benefits from policing interventions, and in what ways, is more challenging. The principal reasons for this difference are that (1) healthcare interventions are, more often than in policing, aiming at treatment rather than prevention; (2) healthcare interventions are more often applied to specific individuals, even when they are preventative; and (3) the direct action on the individual of healthcare interventions makes the mechanism and expected effects more predictable. Thus, the causal connection between medical interventions and their outcomes is usually clear, and the specific individuals benefitting usually identifiable.

Policing interventions, in contrast, often have diffuse and indirect effects. For example, a crackdown on drug trafficking might reduce the availability of drugs in a community, which could have a range of positive effects on public health and safety. However, these effects are difficult to measure and the number of people who directly or indirectly benefit will be hard to estimate. The effects of policing interventions can also be delayed or uncertain. For example, increasing police patrols in a crime hotspot may immediately reduce crime. However, the longerterm effects of the intervention on crime rates, and on the broader social and economic factors that contribute to crime, may not be clear for some time. Policing interventions can also have unintended consequences. For example, intensive stop-andsearch activity might reduce crime in the short term, but they could also undermine community trust in the police,^45^ and lead to social unrest such as the 1981 Brixton riots.

A final, related theoretical point connected to the diffuse nature of many of the benefits of policing services and programs is as follows: we might more naturally understand policing benefits to accrue to communities or other collectives rather than specific individuals. Although it could be possible to put a number on the population size of a geographic community, it would make more conceptual sense to understand these as benefits at the community level rather than as the benefits for each individual member of that community. This is in contrast to the benefits of most healthcare interventions, which accrue directly to the individuals treated: treatment of an individual usually results directly in a health gain for that (and only that) individual. We pick up this point later when we discuss related implications for estimating the “divisibility” of benefits and for estimating “need.”

In summary, we have argued that the benefits of policing are much more diverse in their nature than the benefits of healthcare, and that their incommensurability makes it conceptually incoherent to measure them on a single metric. Even if we accept that the use of QALYs results in an oversimplification of the benefits of healthcare (such that they are not completely suitable in that context either), our point remains that the principal benefits of policing activities are too diverse and incommensurable to be measured using anything like a QALY approach.

In addition to conceptual barriers, there are also greater practical difficulties with measuring the precise effects and reach of policing interventions, as the causal path from intervention to outcome is often far more convoluted and multifactorial, and some diffuse benefits accrue to communities rather than individuals *per se*.

### Difference Two: The marginal benefits of policing programs are likely to be non-linear more often than is assumed in healthcare, making it difficult to estimate costeffectiveness ratios

**Figure 1:**
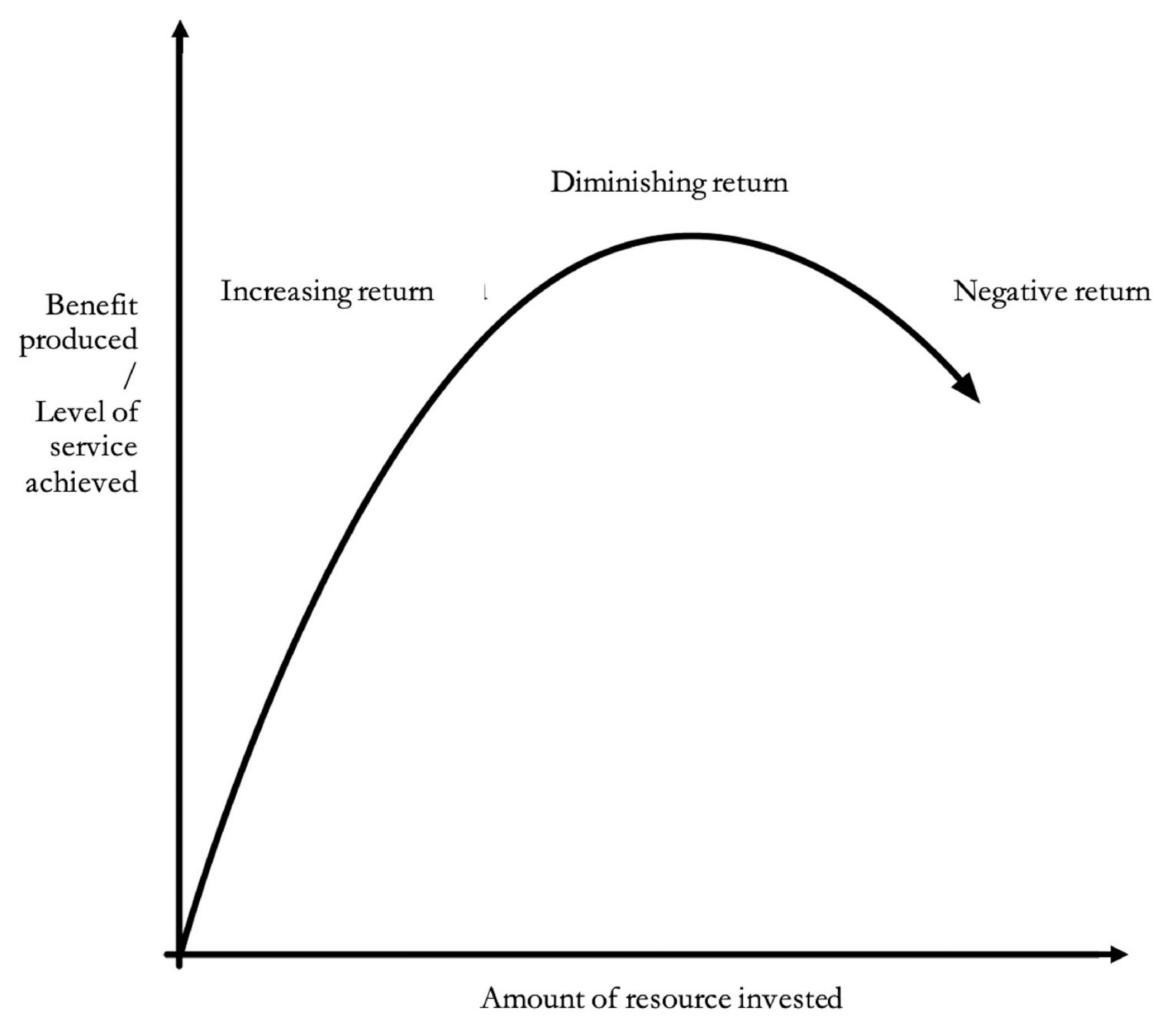
Graph depicting diminishing marginal returns on investment.

Non-linearity of returns to investment can occur when there are diminishing marginal returns. In other words, as more resources are invested in a particular intervention, the additional benefits—marginal utility—gained from each additional unit of investment may begin to decrease. This can occur because of various factors such as negative externalities, saturation of demand, or decreasing efficiency (Figure 1).

Cost-effectiveness analysis of healthcare programs typically assumes that there are linear returns to scale because it assumes that the marginal benefit of each additional unit of healthcare service is constant,regardless of the scale of the program.^46^ To be clear, by “scale,” we are not talking about extent of treatment per individual patient treated (e.g. dosage for a patient), but the number of patients treated. This assumption—that as more patients are treated, the amount of benefit produced increases linearly—is somewhat plausible for some healthcare interventions. For example, the benefit of a treatment program is generally assumed to be the same for each individual who receives the treatment, regardless of the size of the program. Even though there will be individual differences in the magnitude of benefit, approaches that use QALYs take an average estimate of benefit. Models then assume that scaling programs up (e.g. to treat more patients) will not affect the cost-effectiveness ratio (CER) of the program, as the variance in the individual treatment response will remain constant. This permits calculation and comparison of cost-effectiveness ratios of different programs to determine which produces more QALYs per pound.^47^

If the returns on investment are linear, meaning that we get the same health benefit per pound spent, regardless of how much we spend, then comparing interventions based on their CERs is straightforward. We can simply choose the intervention with the lowest CER, as it provides the most health benefit per pound spent.

However, if the returns on investment are non-linear, then comparing interventions based on their CERs becomes more difficult. This is because the CER may change as we scale up or down the intervention. For example, an intervention may be very cost-effective when implemented on a small scale but become much less cost-effective as we try to implement it on a larger scale, or vice versa. In such cases, comparing interventions based solely on their CERs would be misleading. Regardless of whether linearity in returns to scale is a reasonable assumption in healthcare (which it sometimes may not be),^48^ we now argue that this is not a reasonable assumption for policing programs, the majority of which cannot be assumed to be linear in their returns to scale.

Indeed, empirical criminology evidences non-constant returns in policing, meaning that additional resources yield diminishing benefits after a point. This is seen both in overall force size and specific interventions like hot spot policing. Drake and Simper showed mediumsized forces as more scale-efficient than the largest or smallest ones, indicating a “saucer-shaped” average cost curve in policing.^49^ Similarly, Stockdale et al. found that, as police services grow beyond a certain size, they typically face diseconomies of scale, reduced efficiency, or both.^50^

Non-linear resource-benefit relationships also exist in officer count and/or efforts. The Kansas City Patrol Experiment found that saturating areas with police does not further cut crime or boost arrest resolution rates.^51^ Koper demonstrated optimal patrol stops at crime hotspots need around 10 min for maximum residual deterrence, with 11–15 min as optimal stop length.^52^ Beyond this, continued police presence sees diminishing returns.

Therefore, healthcare’s cost-effectiveness analysis methods, assuming linearity, are not fit to guide policing resource allocation. Determining when spending in a specific area reaches diminishing returns opens up possibilities for scaling down or “partially funding” police programs without necessarily losing much benefit. For instance, a scaled down resource like a single patrolling officer in a defined area can still yield significant crime reduction. We argue in the following section that, due to both benefit maximization and equity reasons, policing programs have greater “ethical divisibility” than healthcare programs.

### Difference Three: There is greater prospect for ethical divisibility (permissible scaling down) of programs in policing than in healthcare due to the increased divisibility (shareability) of resources

In general, PBMA results in healthcare programs either being funded in their entirety or not at all. In contrast, we have argued that efficiency considerations will sometimes recommend “partial” or scaled back funding for policing programs, due to greater incidence of non-constant returns to scale. We will now argue that partial funding of policing programs will often also be more ethically permissible than partial funding of healthcare programs.

First, we draw a distinction between *technical* and *ethical* divisibility of programs, and explain how the divisibility of resources—the shareability of the benefits produced by a program—bears on its ethical divisibility. In cost-effectiveness analysis, the term “divisibility” refers to the ability of a program or intervention to be implemented at different scales or levels of intensity. Specifically, a program is said to be divisible if it can be delivered in smaller or larger units, with the cost and effectiveness scaling proportionally. This is *technical divisibility*. For example, consider a program aimed at reducing the incidence of a disease. If the program is divisible, it can be implemented on a smaller scale, such as in a single clinic, or on a larger scale, such as across an entire region. In contrast, an example of an indivisible program might be building a new hospital, which will require a significant investment of resources upfront and may not be easily scaled down.

Due to the nature of their delivery, the majority of programs in policing and healthcare are likely to be relatively technically divisible and permit partial funding. In policing, for example, the number of police officers deployed to a specific area may be scaled up or down. Although the benefits may not scale proportionality, the point remains that the program itself is divisible and permits partial funding. Moreover, both healthcare and policing services are typically delivered at multiple levels, from local clinics and police stations to regional and national levels. This hierarchical nature of service delivery also supports the ability to scale interventions up or down, making them more divisible and technically permitting partial funding.

In distinction to the *technical divisibility* of programs, we can consider their *ethical divisibility*. A program is ethically divisible if it is morally permissible to partially fund it or scale it back. Even if a program is *technically divisible*, it may not be *ethically divisible* if it means some individuals in equal need are denied equal access to the same treatment or service (so failing to achieve horizontal equity). Whilst assessment of equity will of course require consideration of services in combination (an alternative program might provide the same benefit to those not provided for by the scaled back program), the point remains that, in general, scaling back a program raises questions about equity. Sendi raises this concern and illustrates: “a program might also be indivisible because of ethical reasons. It might be seen as unethical to provide some ‘old’ therapy to a fraction of the patients while the other patients receive the better ‘new’ therapy.”^53^

Many healthcare programs will be ethically indivisible. Although it might be ethical to fund a program for certain patient groups only (for example, COVID booster vaccinations for those over a certain age), if a program is funded, it must be accessible to all patients in that patient group, for the geographic region in which it is offered. So, even though a vaccination program could technically be partially funded to provide vaccinations for, say, half of those over the age of 65, ethically it should not, as this contravenes a duty to provide the same services to those in equal need. If a healthcare program cannot be fully funded, it may be ethical to stratify patients on the basis of disease severity or prognosis, to then fully deliver a “sub-program.”

We now explain why, in contrast, many policing programs will be ethically divisible, as a consequence of benefits being more shareable (i.e. greater *divisibility of resources*). This coheres with our earlier point that many benefits accrue to communities, not specific individuals.

Policing activities are often designed to target specific areas or communities, such as high-crime neighborhoods. The benefits of these programs are shared by all members of the community, rather than accruing to specific individuals. For example, increased police patrols and community engagement programs can lead to reduced crime rates and increased feelings of safety, which can benefit everyone in the community. Accordingly, policing resources are to some extent “divisible,” as they are shared over areas or communities. Even a police officer responding to a specific burglary may be able to prevent additional burglaries in the same neighborhood simply by being present and visible.

Conversely, healthcare interventions are often designed to treat individual patients. While the benefits of effective healthcare treatments can be significant for individuals, they are not typically shared beyond the individual (vaccinations contributing to herd immunity would be an exception to this). This is in part due to the indivisibility of healthcare resources: specific resources like drugs or individual medical devices, at least, cannot be divided among multiple patients. While many people benefit from having an officer patrol their streets, or from the apprehension of a prolific burglar, two patients cannot share a single pill.

Consequently, scaling back policing programs does not straightforwardly generate inequity between specific individuals, as the loss is shared across the community. Of course, concerns regarding equity might arise when looking at levels of funding across *different communities*, but the point remains that *within a community*, partial funding of policing programs is less ethically problematic because the burden of the (risks of the) funding cut is distributed across the community, rather than being borne by specific individuals. This is in contrast to healthcare, where scaling back programs can result in specific individuals certainly being denied equal access to medications or treatments.

To recap: we have argued that healthcare programs, although often technically divisible, are less ethically divisible than policing programs, due to the fact that healthcare resources are often indivisible, and their benefits not shared across individuals. This is in contrast to the greater divisibility of policing resources, which often generate benefits that are shared by a community. As such, scaling up and down affects all (potentially benefited) members of the community more equally, rather than resulting in zero-sum situations where a scaled back treatment necessarily cannot be accessed by all in equal need.

In combination with our previous argument that scaling up policing interventions will often result in diminishing returns after some point, we can now conclude that not only does benefit maximization generate moral reasons to optimize the scale of funding of policing programs (which may prompt some scaling back), the increased ethical divisibility of these programs means that scaling back is more ethically permissible than it is in healthcare, in contexts of limited resources.

However, as noted, this does not make it permissible to scale back in the absence of some consideration of need. Scaling up or down must be sensitive to between-community comparisons and relative need for resources (vertical equity). Those in greatest need will have a greater claim on the limited resources. Whilst this is true for resourcing in both healthcare and policing, we will now argue that the way that need should be estimated differs between the two sectors.

### Difference Four: Assessment of “need” in healthcare and policing will be different, particularly as the focus is more often on individuals in healthcare but collectives in policing

The Chairman of The Royal College of General Practitioners, Sir John Toby, stated: “We don’t believe there should be discrimination on any grounds other than on clinical need.”^54^ This aligns with the principle of vertical equity.^55^ However, how “clinical need” should be conceived is a matter of debate, and there is no formal definition.

“Capacity to benefit” has been proposed as a measure of need for resource allocation in healthcare.^56^ Estimating the counterfactual for individuals is a crucial aspect of this, i.e. estimating what would happen to the patient if they did *not* receive the treatment vs. if they did.

One of the challenges of using “capacity to benefit” as a measure of need in policing, however, is that estimating counterfactuals for specific individuals is much more difficult compared to healthcare. For instance, in healthcare, we can estimate with some confidence the negative health outcomes a person with a medical condition may suffer without treatment. However, in policing, it is much harder to know if a particular individual would have been a victim of a crime without increased police presence, although statistical likelihood of victimization could be a very rough guide. Counterfactuals are easier when it comes to investigative activity, as the default counterfactual will be an unsolved crime. However, in general, any measure of need that required the likely *individual* benefit to be a determinative factor would not work well for policing, due to too much counterfactual uncertainty.

Second, even if it were possible to make rough estimations of individual-level benefit from policing resources, we do not think this is the correct conceptual unit of analysis. We argued above that, since it makes more sense to conceive of the benefits of policing as accruing to collectives, it correspondingly makes more sense for the “need for resources” to be considered at the same level.

As we elaborate in the discussion below, a more conceptually coherent and practicable approach to allocating policing resources would be to consider the needs of collectives, such as geographic communities, in terms of burden of crime, public safety gaps, and vulnerability. We prefer the term “collectives” to “communities” as some relevant groups— such as vulnerable children or mental health patients—will not constitute a community in either the geographic or social sense of the word.

### Difference Five: Considerations of need will more clearly trump efficiency in the policing context

In addition to arguing for the assessment of need at the level of collectives, we also propose that reasons to distribute resources according to “need,” as a way of achieving a vertically equitable distribution, will more clearly trump (although not eclipse) efficiency considerations in policing. This will have consequences for the relative weight that decision-makers should give to these factors, and the way that trade-offs are resolved. Our argument for this proposal is based both on normative reasons, relating to the legitimation of the policing service, and on instrumental reasons relating to the positive effects of perceived legitimacy on cooperation and compliance.

As embodied in the Peelian Principles of policing, the legitimation of the police service is derived from “the consent” of the public it polices, rather than from the mere imposition of coercive power. This aspect of the justificatory basis differs from that underpinning the health service. So, for the operation of the police service to be justified at all, it must be considered acceptable by the public. More precisely, Bottoms and Tankebe propose that legitimate policing requires “congruence between the system and the practices of policing, on the one hand, and the societal beliefs, values and expectations that provide its justification, on the other.”^57^ By allocating resources on the basis of need, the police service demonstrates a commitment to fairness and that the police are dedicated to protecting the most vulnerable and marginalized members of society. Bottoms and Tankebe underscore the necessity of fairness for justified authority as follows: “groups who experience either over-enforcement or under-enforcement of the law tend to blame the police for what they have experienced, so this often leads to a diminution of attributions of legitimacy. Accordingly, distributive fairness is an appropriate and necessary element in the police’s response to the public’s basic legitimation demand.”^58^

In addition to distributive fairness being necessary for legitimacy per se, allocating resources based on need can foster positive relationships between the police and the public, which also serves an instrumental value. When individuals perceive the police to be fair, this can lead to increased cooperation with law enforcement efforts.^59^ The public is more likely to engage with the police, share information, and actively participate in crime prevention initiatives when they feel that their needs are being taken seriously. Adopting a principally need-based strategy for allocating resources is therefore likely to also have positive consequences for the effectiveness of the whole service, which functions better with greater public cooperation.

Conversely, prioritizing efficiency above “need” risks alienating communities and eroding trust in the policing service. Directing resources disproportionately towards areas with low crime rates or other privileged factors can breed resentment and perpetuate social disparities. This not only undermines the legitimacy of the police service per se, but is also likely to lead to decreased cooperation, community engagement, and a weakened sense of collective responsibility for safety.

In contrast, the justification for the healthcare service (as opposed to specific medical interventions) does not rely on public consent, as it does not (for the very most part) assert coercive authority. Nor does it rely on the cooperation of the public to the same extent in order to serve its function. This has the consequence that the healthcare service can pursue the goods of aggregate benefit maximization more equally alongside considerations of need, even where this results, for example, in not funding relatively inefficient interventions for severe illnesses. In contrast, although the police service should consider the most efficient way to respond to a severe need, that this need generates a claim on resources is the factor that looms largest.

## Discussion and Implications

Given the differences we have identified, and the lack of existing proposals for ethical resource allocation in policing, a novel decision-making approach needs development. Although we lack the space to develop such a proposal here, in this final section we further draw out the implications of the differences examined above, and tentatively sketch some ideas for an ethical policing resource allocation decisionmaking framework. This framework, we envisage, would be used on a repeated basis, at least annually, and certainly whenever there is a significant change to relevant factors. This will ensure that resource deployment is reviewed as budgets and circumstances change.^60^ We are not envisaging a “one off” resource allocation exercise. Indeed, monitoring and review will be essential to ensure that allocations remain the most ethical. Relatedly, we envisage this framework to facilitate medium-to-long-term, “day-to-day” resource deployment, i.e. to departments, local areas, and specific police functions etc. This does not preclude that on occasions resources will be redeployed out of those functions to tackle specific immediate, urgent problems, such as riots.

To recap, based on our arguments, a resource allocation framework for policing must have the following features: Vertical equity—a measure of “need” for resources—should feature as the primary allocative consideration, and should be assessed at the level of collectives. Although efficiency will feature as a further, important consideration, cost-effectiveness ratios cannot be used as they rely on commensurable benefits and linear returns to scale. Efficiency must therefore be assessed by comparing the magnitude of improvements (or reductions) to levels of service for a given cost (or saving), seeking to avoid significant opportunity costs, and identifying opportunities for partial funding.

We now propose a formula and stepwise procedure that incorporates these dimensions. Our aim, at a general level, is to enumerate the relevant conceptual features and their approximate relative influence on resourcing decisions. Although we will then make some suggestions for how these features could be operationalized and measured, we do not set out to fill in all the details at this time.

Our stepwise proposal is as follows (Table 1):

Steps 1 and 2 relate to the estimation and prioritization of “need” as an allocative factor. Step 3 relates to the assessment of efficiency and measuring benefits. Steps 4–6 relate to the procedural principles we outlined earlier in the paper. We now expand on steps 1 and 2 and estimations of need.

### Approach to Estimating the Relative “Need” for Resources

We argued above that need should be the primary consideration when allocating policing resources, with efficiency considerations guiding finergrained allocation once need-based priorities have been earmarked. Our proposal makes need the first, and most influential factor.

A comprehensive framework would have to provide further analysis of what constitutes “need” in this context. Some existing work in policing has conceptualized “need” as “demand,”^61^ which we suggest overlaps with but is not identical to the concept of need. Demand is a concept best captured by the idea of simply *making* claims on resources, rather than *having* a claim on resources.^62^ Those making the loudest claims on resources may not actually need them the most. For example, a policing area might get more crimes reported than another area, but if many of these are relatively low level, then that greater demand does not necessarily mean there is greater need. Although demand will remain an important concept in service delivery, allocation of resources should be primarily concerned with the degree of “need” of various collectives, such as geographic areas, or population groups such as vulnerable children, the elderly, or mental health patient groups.

We propose that the degree of need of a given collective is determined by the incidence and seriousness of the crime to which it is (or is likely to be) subject, and/or the magnitude of harm caused by gaps in public safety functions (such as traffic policing, licensing of alcohol, crowd control) that serve the collective. We also propose that an estimation of crime incidence and public safety gaps is magnified by the degree of vulnerability of those constituting the collective, to get a full estimation of the degree of need. This is because the same types of crime can have differential impacts depending on initial vulnerability, and because vulnerability itself generates a claim on public resources that are intended foremost to protect, and which can to some extent mitigate or alleviate the consequences of that vulnerability.

We therefore propose that the conceptual aspects of a formula to estimate magnitude of need are:

$$\begin{array}{l} Need\;=\\ ({(}_{\text{Reported}}{+}_{\text{Unreported}}\text{Crime-Harm}\;\text{Rate}\\ +\;\text{Public}\;\text{Safety}\;\text{Gaps}\;)\\ \times \;\text{Vulnerability}\;) \end{array}$$

where “_Reported + Unreported_Crime-Harm Rate” is a measure of the aggregate harm to a collective from reported and unreported crime. Including the rate gives an indicator of the scale as well as the severity of the harms.

“Public Safety Gaps” is a measure of the severity and likelihood of harm predicted to result from the public safety gap(s). This captures other, non-crime needs, such as missing persons, road safety etc.

“Vulnerability” is a measure of how vulnerable the affected community or group is. The College of Policing defines a person as being vulnerable “if as a result of their situation or circumstances, they are unable to take care of or protect themselves or others from harm or exploitation.”^63^

How might “need” be estimated in practice? We do not have space here to offer a detailed proposal. However, it is entirely plausible that a mixture of tools and data could be used to generate rough estimations of harm-indexed crime incidence, and of actual or predicted harms from gaps in public safety functions. For example, The Cambridge Crime Harm Index (CHI) is a tool developed by researchers at the University of Cambridge to measure the severity of harm caused by different types of crime.^64^ The CHI assigns a harm score to each reported crime based on the severity of the crime as indicated by the number of days imprisonment an offender would serve if they received the “starting point” for sentencing. However, the CHI will not provide all the data needed. Many crimes are not reported, so a measure like this should be supplemented with intelligence and national surveys on the unreported incidence and impact of crime, to provide an overall estimate, which we have designated as _R+ U_C-HR. To standardize weighting, a score, with a range perhaps from 1−5 (low-severe), could be allocated.

Similarly, data on the actual and likely harms caused from gaps in public safety functions (non-crime functions of the police service), such as traffic policing, could be used to assign a score (perhaps from 1-5) to indicate the severity and scale of the problem.

Forces may use demographic data, such as information on poverty rates, education levels, and access to health care, to identify vulnerable populations that may be disproportionately affected by victimization or may be in greater need of protection. Census data would be once such source. By directing resources to these populations, such as through community outreach programs or targeted enforcement efforts, the need for earlyintervention can to some extent be met, and faster response rates achieved. We propose that an estimate of level of vulnerability, perhaps 1−3, could then multiply the crime-safety burden (_R+ U_C-HR+ PSG) to provide an overall comparative measure of need for resources.

Whether the crime burden is scored from 1−5 or 1−7, for example, will affect the weight it has in the formula, and whether the severity of the public safety gaps is scored on a smaller, equal, or larger scale, will affect the relative weighing for these factors. The same is true of the scoring of vulnerability: the larger the scale, the greater the impact on the overall Need score. We suggest that Rawls’ approach of wide reflective equilibrium could offer a method to refine the relative weighting of the factors within the formula.^65^

Reflective equilibrium involves a process of iteratively adjusting principles and judgments to achieve the most coherent and therefore most ethically justified framework. Applying this method to assign the relative weighting would involve iterative deliberation and adjustment among stakeholders, including policing policymakers and communities. In the UK context, this would include the force’s Chief Constable and the Police and Crime Commissioner, who represents the public of the force’s area. It would also include input from marginalized groups or those otherwise without a voice, e.g. through independent advisory groups or community stakeholder groups. The process might commence by using clear examples where the level of resource need, at least comparatively, is unambiguous (e.g. cases of very high or very low need), to calibrate the formula. These examples would serve as reference points, enabling the formulation of initial weights for different factors. With the calibrated formula, more challenging cases could be assessed more confidently, as the preliminary calibration would provide a foundation for making prima facie judgments. Through making iterative adjustments in response to the practical implications of the chosen weights for different factors, an ethically justified resource allocation formula could be developed. This process would ensure that the relative importance assigned to each criterion achieves the best possible coherence between theoretical principles, judgements about fairness, and practical considerations.

Of course, not all funding is directed to the front line. Police forces are organizations with extensive administrative, managerial and other support functions. Laufs et al. distinguish front-line policing demand from organizational demand.^66^ In a full review of a force’s allocation of resources, these areas of spending would also have to be examined to ensure that, overall, the distribution of funding was justified. We suggest that the degree of need for these organizational services is contingent on the degree of need for the front-line police services they support. If there is a greater need for policing services in a particular area or department, the workload for administrative and other support staff will increase as well. Organizational need is therefore an instrumental ethical consideration, to the extent that it increases the efficiency of the policing activity addressing crime and public safety needs.

In addition to organizational spending being necessary to directly support the effectiveness (and therefore efficiency) of frontline services, there is also a need for further organizational functions which curate the data necessary for assessing the need for frontline services, and for monitoring the efficiency of these services. The police can only achieve an efficient resource deployment if they know, for example, whether services have reached a point of diminishing returns. To know this, substantial data sets are required, and resources dedicated to analysis. In other words, ethical resource deployment requires a fairly substantial “back office” to be able to know how effective “frontline” deployments are, and when the marginal returns are cutting in, as well as to provide the data supporting estimations of relative need for resources. The justification for spending on these functions will not derive either directly or indirectly from the “need” formula above. Rather, separate consideration must be given to resourcing this function, which supports the whole enterprise of allocating resources ethically.

Once a rough order of priorities has been estimated based on need, we suggest that efficiency considerations should fine-tune and direct the allocation of resources to these priorities.

### Approach to Estimating Efficiency and Measuring Benefits

Steps 3 and 3.1 relate to how efficiency should be factored into addressing priorities. Before suggesting *how* efficiency could feature in allocative decisionmaking, we must suggest how efficiency could be estimated. The approach we sketch here would allow decision-makers to make *some* estimation of the relative benefit of funding different activities in policing, without needing to be able to compare the precise magnitude of benefit on a single metric.

We have proposed:

$$\begin{array}{l} Efficiency\;=\\ \;(\text{Improvement}\;\times \;\text{Public}\;\text{Value)/cost}).\; \end{array}$$

NB. Improvement X PV captures the *expected benefit* of a proposed intervention.

NB. This provides a measure of efficiency of the intervention *at the particular scale of implementation*.

Where “Improvement” is a measure of the increase in the level of service expected to be provided by the intervention. “Public Value” represents an estimate of the public value of that service, taking into account its societal importance. Together, “Improvement” and “PV” quantify the potential positive impact or value generated by the intervention: i.e. the “Benefit.” “Cost” represents the expenses incurred in implementing the intervention or project at the proposed scale. It includes all relevant costs, such as resources, personnel, materials, and ongoing maintenance.

Although we do not have the space to fill in the details here, at the conceptual level, we suggest that an assessment of efficiency—or “value for money”—could take into account the rough magnitude of the improvement (or impairment) in valuable service that would be gained from a particular investment (or saving). This would give some indication of value for money of a particular funding proposal at a particular scale of implementation. Magnitude of improvement (or impairment) could be estimated using descriptors, e.g. a score of 1−5, from minimal improvement to transformational improvement, with careful wording to help decisionmakers assess the expected magnitude of improvement. Crucially, this estimation would not yet attach value (i.e. an estimation of worth) to this improvement, merely signal if, for example, the proposed investment would make much of a difference to the particular area of service delivery, in terms of things like time taken, number of cases solved— essentially the *size of the change to effectiveness*.

This descriptive assessment must be supplemented with an evaluation of the *importance* of the area of service within which the change would occur to get a sense of the overall expected benefit. A large improvement to an area of lower public value should not necessarily be considered more beneficial than a small improvement to an area of high public value. For example, reopening more front desks in a neighborhood may be a substantial improvement in terms of the functioning of that area of service, but it may be of lower public value compared to a service area such as murder investigations. Even a modest improvement there could be highly valuable, and more valuable than the substantial improvement in front desk function. This is why we suggest a Public Value multiplier. As above, a reflective equilibrium approach could be used to calibrate the relative weighting of “Improvement” and “Public Value.”

Although a full account of the public value of an activity would need to be developed and operationalized for policing, as its core, a measure of public value is going to capture the extent to which an activity advances and protects the interests of the public.^67^ We suggest that something like Moore and Braga’s public value scorecard could be adapted for this purpose, where areas of policing service are scored relative to how many of the valuable functions they serve. For example, a score card might include “Crime prevention,” “responding to crime,” “investigation of crime,” “reassuring communities,” “addressing public commitments and local priorities,” “expanding access to services (horizontal equity).” Weightings could be given to these values, as well as scope for areas of service to serve these to a greater or lesser extent and serve more than one. The scorecard would thus provide a way of estimating the public value of the *type* of activity, but not the total value a specific improvement in the activity actually produces. This total value is estimated by multiplying the expected improvement in the activity (essentially its increased effectiveness) by its public value, to estimate overall how much value it produces.

This approach incorporates both the objective improvements in service level and the subjective evaluation of the value of the benefits, which should be determined in consultation with stakeholders. When divided by the cost of a proposed investment, decision-makers are given an indication of the value for money at that scale of implementation. *Loss* of value for a proposed *saving* can be estimated in the same way using a mirrored scale for size of impairment to a service, multiplied by its public value. It may be that some service areas have reached a point of generating only minor improvements for considerable costs, which could indicate opportunities for shifting funds to areas that could generate more substantial improvements for the same money.

Thus, as set out in the framework above, once the priorities have been identified on the basis of need, decision-makers can use these value-for-money or loss-of-value-for-saving estimations to compare possible ways to attend to the top priorities in terms of relative efficiency, with partial funding considered to optimize and attend to more priorities.

This approach overcomes the incommensurability problem because, even though we do not want to say, for example, that preventative benefits are more important than investigative benefits (which may include deterrence, retributive value, support for victims), because of course both are valuable, decision-makers can still see if an area accruing investigative benefits is well over a minimally sufficient standard or even at diminishing returns compared to an area that could produce a lot of extra preventative benefit (as it is still in the increasing returns zone).

Put another way: we do not have to say whether apples are absolutely better than oranges (such that apples should always be produced over oranges): if one allocation of resource produces barely any extra apples whereas a different allocation of that same resource produces a lot of oranges, then that is likely to be a better use of resources (especially if it has been identified that there is a higher need for oranges). We can be quite confident in such a situation that more good is being done with this allocation, even if apples and oranges cannot directly be compared in value.

Although the proposed approach is much cruder than traditional costeffectiveness analysis, we believe that something like this would capture the relevant factors, with the right order of importance, and permit rough comparisons that improve on the status quo approaches to resource allocation in policing.

### Other Elements of the Framework

In developing a framework, decisionmakers will have to also factor in any other grounds for allocating resources, such as promises made to communities, responding to public campaigns and requests (for example, to address less acutely urgent but long-standing problems), national directives, and also some statutory functions that the police are required by law to resource regardless of the ethical weight of doing so, e.g. freedom of information requests, provision of data barring service information, authorizing of data requests under RIPA, etc. We suggest that these are often of lower ethical weight than need and efficiency, but still are important. Thus, in step 4, as part of the iterative review, these considerations are brought into the mix and adjustments made.

### How This Differs from Programme Budgeting Marginal Analysis

Above, we introduced PBMA, an approach used in healthcare, which incorporates Multi-criteria Decision Analysis (MCDA). MCDA allows a program to be scored on various factors, which may be weighted in importance. Although our approach shares some similarities, particularly in enumerating multiple factors and ascribing differing degrees of importance to them, we believe that our “nested” approach is conceptually neater, with clear relations *between* factors (and aspects of these factors), which improves justification and conceptual clarity. For example, in the proposals drawn up by Wilson et al. for healthcare, some of their criteria overlap: the criteria of “Effectiveness,” “Prevention” and “Quality of life (QALYs)” will arguably overlap and result in some double counting, without clear distinctions and relations between these aspects.^68^ In our framework, in contrast, we have nested magnitude of improvement and public value within benefit, and have ensured that they are specified such that they do not overlap. We have also nested a number of aspects within public value, comprising the various valuable goals of policing, with a degree of weighting. Our approach also differs by being nested in a stepwise fashion, with need providing a suggestive but not definitive initial order of priority, rather than being one of the several, weighted criteria that exert their influence on the score simultaneously.

MCDA also differs in its use of scalable cost−benefit ratios to generate a list, and a binary approach to funding decisions from the top of that list until funds are expended, which we have argued is not appropriate for policing. Instead, we have proposed using estimates of a program’s value for money at a particular scale of implementation (i.e. using non-scalable benefit−cost ratios) and comparing programs at different scales of implementation.

Despite these differences, both approaches attempt to capture and systematize the various relevant factors that go into making resource allocation decisions. We are sure that our proposals will face challenges too. We wholeheartedly agree with Wilson et al. when they say “we have emphasized that the rankings generated by our tool should be seen as a starting point from which meaningful discussions can take place to decide priorities, and are not the solution itself.”^69^ Indeed, in our approach, we have avoided trying to achieve the precision of definitive rankings and left more room for decision-makers to adjust as they go through the steps. Achieving the ideal balance of technical precision and room for judgement will be a significant challenge in developing our ideas further.

### Applying the Framework

There is much detail that needs filling in before our framework can be fully applied. However, we can already start to see some of the implications for the considerations at play in the scenario at the beginning of our paper. Of course, a Chief Constable would have many additional areas of spending to review when making decisions, but we can see, for example, that it is likely that the decision to close the front counter to make savings would be supported by our framework, assuming that it does not do much to respond to a crime or public safety need (so there is low need for it), and even if reopening would be a substantial improvement to the status quo, this improvement would be of relatively low public value, based on the scorecard we envisage (so low or moderate efficiency, depending on cost). Unless there has been a public commitment to reopen the front counter, any further considerations in step 4 are unlikely to move this to a high priority.

Our framework is likely to protect child abuse investigation from cuts, particularly given the existing resourcing pressure and sick leave owing to stress and burn out. The _R+ U_C-HR score is likely to be high due to the seriousness of the crime, and the vulnerability of the child victims is also likely to be high, which would result in a high “need” score. Reducing funding any further is likely to significantly reduce the level of this high public value service, especially given that officers are already so overstretched, so any savings proposed here would be unlikely to be the most efficient savings. Further, poor officer welfare—aside from its consequences for service—would factor into additional considerations in step 4.

## Conclusion

In this paper, we have argued that there are fundamental differences between healthcare and policing, which make a difference to the approach that should guide resource allocation. Although, at the general level, maximizing the benefits of expenditure and responding to need will be relevant in both sectors, we have argued that a different approach to comparing opportunity costs and assessing need will be required. We have argued that need should be the principal allocation criterion in policing and assessed at the level of collectives. We have also argued that partial funding of programs is more ethically permissible in policing than in healthcare, and that the greater prospects for diminishing returns may in many cases make scaling back economically prudent too.

To account for these differences and their implications, we have sketched the contours of an ethical resource allocation framework for policing. We have argued that data indicating the severity and incidence of a crimesafety burden, along with data indicative of socio-economic vulnerability, could be used to compare geographic or other collectives in terms of the relative claims they have on resources— their “need.” We have suggested that efficiency should direct allocation after need has been considered as a primary factor. We have suggested that further considerations, such as legitimacy and public confidence should then be considered to confirm or challenge the allocations indicated.

We have sketched some ideas for making assessments of the relative benefits likely to be generated by shifting funds from some activities or programs to others. We have argued that an estimation of the magnitude of improvement to a service area, weighted by public value, could be a fruitful approach for estimating where funding is likely to produce greater benefit. Where funds are being shifted, the possibility of partial or reduced funding should first be considered if the alternative is to cut completely with no comparable substitute program.

Clearly, this is a very preliminary attempt at developing an ethical resource allocation framework for policing. Our primary goal has been to demonstrate the need for considerably more work on this topic and to show that novel approaches will be required. We hope this serves as a springboard for discussion within academia and the police service as to what approaches could meet this urgent need.

## Figures and Tables

**Table 1:** Framework for Ethical Resource Allocation in Policing.

Step 1: Calculate “Need Score” for Each Intervention under consideration (for example, how many additional officers to put into neighborhood policing)	Use a formula that allows estimation of relative need for resources. We propose: Need = ((_R + U_Crime-Harm Rate + Public Safety Gap) X Vulnerability). We explain this below. Assign numerical values to each of these indicators based on their magnitude, according to predefined ordinal categories. Calculate the “Need Score” for each intervention.
Step 2: Generate a List of Priorities Based on “Need Score”	Rank the interventions based on their “Need Score” in descending order. This ranking will represent the *initial* assessment of ethical priorities, indicating areas that require more immediate attention and resources due to the severity of the crime-safety burden.
Step 3: Consider Efficiency to Allocate the Budget	Once the list of ethical priorities is established based on Step 2, the next step is to allocate the limited budget between the areas of funding. Use an Efficiency formula to estimate value for money of the interventions at their proposed scale/costing. We propose: Efficiency = (Improvement X Public Value) / Cost. We explain this below. Prioritize funding for interventions with higher Need Scores. When considering different options for addressing a need, compare the Efficiency scores for any alternative implementation options to identify the most cost-effective approach. For interventions with similar Need Scores, use Efficiency rankings to guide funding decisions, allocating more resources to interventions with higher Efficiency scores (which indicates a greater potential positive impact relative to their costs). If the budget is not enough to fully fund all interventions, consider partial funding options for interventions with lower Need Scores, to ensure that areas with higher Need still receive significant resources. *Recalculate Efficiency score based on the improvement and cost at the smaller scale.* Step 3.1: Avoid Significant Opportunity Costs Throughout the allocation process, carefully consider the potential opportunity costs associated with funding decisions, and whether any areas of funding have reached a point of diminishing returns. This would indicate that they might bear some reduction in funding. Ensure that interventions with lower Need Scores but high Efficiency are not dismissed entirely, as they may present valuable opportunities to achieve meaningful improvements with a relatively smaller budget allocation.
Step 4: Iterative Review	After drawing up a proposed resource allocation based on Step 3, review the proposed allocations for any outcomes that could reasonably be challenged. Consider any additional factors such as national priorities and directives, public demand, legitimacy considerations, and feedback from stakeholders, particularly from marginalized groups or those without a voice, e.g. through independent advisory groups or community stakeholder groups. Revisit and amend the proposed allocations based on any new information or insights gained from this reassessment. Review again.
Step 5: Finalize Resource Allocation	Use the results of the iterative process to make final resource allocation decisions.
Step 6: Ongoing Evaluation and Adaptation	Resource allocation decisions should be subject to ongoing evaluation and adjustment. Regularly review the outcomes of funding decisions and reassess the Need and Efficiency Scores of interventions based on their actual impact and effectiveness.

## Footnotes

1 See HMICFRS, *State of Policing*.

2 See Ruta et al., “Programme Budgeting”; Wilson, Rees, and Fordham, “Developing a Prioritisation Framework.”

3 See Harris, “QALYfying”; Culyer,”Equity.”

4 See, for example, Kleinig, *The Ethics of Policing*; Miller and Blackler, *Ethical Issues in Policing*.

5 See Sunshine and Tyler, “The Role of Procedural Justice.”

6 See Ludvig, Norton, and McLean, “Resource Allocation Processes in Policing,” 7.

7 https://fullfact.org/crime/police-numbers/.

8 See College of Policing, “Estimating Demand.”

9 See House of Commons, “Policing for the Future.”

10 See HMIC, “Policing in Austerity.”

11 https://www.dailymail.co.uk/news/article-4908480/Top-cop-police-makingdifficult-choices-cuts.html.

12 https://www.bbc.co.uk/news/uk-england-norfolk-43573049.

13 https://www.london.gov.uk/press-releases/mayoral/cuts-force-mayor-to-reduce-police-front-counters.

14 https://www.mirror.co.uk/news/politics/police-cuts-leave-only-one-26707761.

15 https://www.independent.co.uk/news/uk/crime/police-cuts-budget-crimes-investigated-public-safety-a8895281.html.

16 https://news.npcc.police.uk/releases/in-austerity-police-must-prioritise-calls-and-may-not-attend-for-all-crimes.

17 https://www.independent.co.uk/news/uk/crime/police-uk-stop-responding-crime-budget-cuts-demand-federation-violence-demand-officers-a8485316.html,N.B. The Police Federation of England and Wales is the statutory staff association for police constables, sergeants, inspectors, chief inspectors and special constables in the 43 territorial police forces in England and Wales.

18 https://www.gov.uk/government/news/national-campaign-to-recruit-20000-police-officers-launches-today#:~:text=Home%20Secretary%20Priti%20Patel%20said,the%20best%20in%20the%20world.

19 See Fleming and Grabosky, “Managing the Demand for Police Services.”

20 https://www.justiceinspectorates.gov.uk/hmicfrs/our-work/article/value-for-money-inspections/value-for-money-profiles/value-for-money-dashboards/.

21 See Fleming and Grabosky, “Managing the Demand for Police Services.”

22 Ibid.

23 See Lister, “The New Politics of the Police.”

24 https://publications.parliament.uk/pa/cm201213/cmselect/cmhaff/144/14412.htm.

25 https://www.theguardian.com/uk/2011/mar/28/frontline-police-moved-into-office-jobs#:~:text=Serving%20police%20officers%20are%20being,side%2Deffects%20of%20budget%20cuts.

26 See Ludwig, Norton, and McLean, “Resource Allocation Processes in Policing.”

27 See Zhuang and Collier, “Resource Allocation Decisions in the Public Sector.”

28 Shumate and Crowther, “Quantitative Methods.”

29 Den Heyer, “Examining Police Strategic Resource Allocation.”

30 Farmer, “Police Resources Allocation.”

31 Charman and Williams, “Accessing Justice.”

32 Wheeler, “Allocating Police Resources.”

33 Carr-Hill, “Developing a Robust Resource Allocation Formulae.”

34 Ibid., 242.

35 See Ruta et al., “Programme Budgeting.”

36 Adapted from Ruta et al., “Programme Budgeting.”

37 Mortimer, “Reorienting Programme Budgeting and Marginal Analysis.”

38 Wilson, Rees, and Fordham, “Developing a Prioritisation Framework.”

39 For discussion, see Harris, “QALYfying.”

40 In this section we use the term “efficiency,” which captures the idea of optimized outputs to inputs. Efficiency involves optimizing resource use to achieve desired outcomes with minimal waste. “Effectiveness” (or efficacy) emphasizes the successful achievement of goals. Cost-effectiveness, the concept employed by decision-making bodies in the UK, specifically evaluates whether resources invested are justified economically in achieving goals. In essence, efficiency is the broader goal, effectiveness focuses on goal achievement, and cost-effectiveness is one particular measure of efficiency, which considers the economic costs of achieving effectiveness (or efficacy).

41 See WHO, “Making Fair Choices.”

42 WHO, “Making Fair Choices,” 8.

43 College of Policing, “Policing Vision (2025).”

44 Miller claims that policing is justified (and that its aims are structured) by its purpose to protect legally enshrined, justifiably enforceable, moral rights (both human rights and institutional rights): rights to life, property, security and so on. See Miller and Blackler, *Ethical Issues in Policing*; Miller and Gordon, *Investigative Ethics*.

45 https://www.college.police.uk/app/stop-and-search/stop-and-search.

46 See Drummond et al, *Methods for the Economic Evaluation*; Muennig and Bounthavong, *Cost-Effectiveness Analysis in Health*.

47 To recap: when conducting a cost-effectiveness analysis, a cost-effectiveness ratio (CER) is calculated for each intervention or program. The CER is the cost of the intervention divided by the health benefit it provides, usually measured in QALYs. For example, let’s say we have two interventions: A and B. Interventions A and B both cost *£*100,000, but intervention A produces 10 QALYs and intervention B produces 20 QALYs. We can calculate the CER for intervention A as *£*10,000 per QALY and the CER for intervention B as*£*5,000 per QALY.

48 See Lord, Laking, and Fischer, “Health Care Resource Allocation.”

49 Drake and Simper, “Productivity Estimation.”

50 Stockdale, Whitehead, and Gresham,*Applying Economic Evaluation.*

51 See Kelling et al., “The Kansas City Patrol Experiment.”

52 Koper, “Just Enough Police Presence.”

53 Sendi, “Some Reflections,” 192.

54 See, Cookson and Dolan, “Principles of Justice.”

55 As a reminder, vertical equity in healthcare resource allocation refers to the principle that resources should be allocated based on individuals’ varying levels of need for healthcare services. This means that individuals with greater healthcare needs, such as those with more severe illnesses or disabilities, should receive a greater share of resources than those with less need.

56 For discussion, see Culyer and Wag-staff, “Need, Equity and Equality.”

57 Bottoms and Tankebe, *Police Legitimacy*, 61.

58 Bottoms and Tankebe, *Police Legitimacy*, 79. N.B. Bottoms and Tankebe in fact argue for a fourfold conceptualization of the potential components of criminal justice legitimacy, as viewed by those subject to state power, namely procedural justice, distributive justice, effectiveness, and lawfulness. We note that although effectiveness is a consideration here, maximizing *cost*-effectiveness (i.e., efficiency) is not amongst their requirements for legitimacy.

59 See, for example, Bradford, “Policing and Social Identity.”

60 Many factors will change how resources should be allocated. These include fluctuating crime rates, changes to the costs of delivering particular areas of business (e.g., when new technology allows certain functions to be delivered more cheaply or effectively), changing public views, and revised national priorities, amongst many other fluctuating factors.

61 Laufs et al., “Understanding the Concept of ‘Demand’ in Policing.”

62 See Culyer, “Equity.”

63 https://www.college.police.uk/guidance/vulnerability-related-risks/introduction-vulnerability-related-risk.

64 See Sherman, Neyroud, and Neyroud, “The Cambridge Crime Harm Index.”

65 Rawls, *Justice as Fairness*.

66 Laufs et al., “Understanding the Concept of ‘Demand in Policing.”

67 Moore, “Creating Public Value”; Moore and Braga, “Measuring and Improving Police Performance.”

68 Wilson, Rees, and Fordham, “Developing a Prioritisation Framework.”

69 Wilson, Rees, and Fordham, “Developing a Prioritisation Framework,” 8.
